# Prevalence and phenotypic characterization of carbapenem resistance in multidrug-resistant Gram-negative bacteria across selected healthcare facilities in the United Arab Emirates: a retrospective study

**DOI:** 10.1186/s12879-026-13007-0

**Published:** 2026-03-13

**Authors:** Rula A. M. Aljaede, Semira Abdi Beshir, Ammar Ali Saleh Jaber, Ramadan Elkalmi, Eiman Shamseldin Al Gailani Ali, Amany Hamed Mahmoud Khedr, Dujana ALHamed, Jainamboo Meera, Zulfa AlDeesi

**Affiliations:** 1 Department of Pharmacy Practice, College of Pharmacy, Dubai Medical University, Muhaisnah 1, P. O. Box 19099, Dubai, United Arab Emirates; 2 Faculty of Health Sciences, Higher Colleges of Technology, Dubai, United Arab Emirates; 3 Department of Pharmacology, Faculty of Medicine, Sebha University, Sebha, Libya; 4 Department of Microbiology, Thumbay Laboratories, Ajman, United Arab Emirates; 5 Department of Infectious Diseases, Rashid Hospital, Dubai Health, Dubai, United Arab Emirates; 6 Department of Microbiology, Rashid Hospital Laboratory, Dubai Health, Dubai, United Arab Emirates; 7 Microbiology and Infection Control Unit, Latifa Hospital, Dubai Health, Dubai, United Arab Emirates

**Keywords:** Carbapenem resistance prevalence, Multidrug resistant Gram-negative bacteria, Carbapenem-resistant *Enterobacterales*, Carbapenem-resistant *Acinetobacter*, Carbapenem-resistant *Pseudomonas aeruginosa*, Carbapenem-resistant *Klebsiella pneumonia*, Infection control and surveillance, COVID-19 outbreak, Healthcare associated infections

## Abstract

**Background:**

Carbapenems are essential antibiotics used to treat severe nosocomial infections. However, the misuse of these antibiotics has led to the emergence of carbapenem-resistant organisms (CROs), posing significant public health threats. This study aims to determine the prevalence and phenotypic profile of carbapenem resistance (CR) in multidrug-resistant Gram-negative bacteria (MDR-GNB) in selected healthcare facilities in the United Arab Emirates (UAE).

**Methods:**

A retrospective study was conducted across 65 healthcare facilities in Dubai and the Northern Emirates (NE) from January 2018 to June 2021. Results from GNB isolates - including *Enterobacterales*, *Acinetobacter*, and *Pseudomonas aeruginosa*- obtained from various clinical samples were collected and assessed for CR using CLSI guidelines at all locations. MDR-CRO prevalence was calculated within each bacterial group using total isolates of that group as the denominator at each time point.

**Results:**

Of the 77,228 GNB collected, 3,829 (5%) MDR-CROs were identified. Within the MDR-CROs subset, *Enterobacterales* accounted for 65.8% (48.7% of which were *Klebsiella pneumoniae*), *P. aeruginosa* 28.6%, and *Acinetobacter* 5.6%. In contrast, period prevalence calculated within total isolates of each organism group was highest for MDR-CR *Acinetobacter* (22.8%), followed by MDR-CR *P. aeruginosa* (12.9%), and MDR-CR *Enterobacterales* (5.6%). A significant increase in MDR-CR *Acinetobacter* was observed in the NE in mid-2021, which coincided with the COVID-19 pandemic. Hospitals were the primary source of MDR-CR isolates, accounting for more than 90% of *Acinetobacter* and *P. aeruginosa* cases, and 62.6% of *Enterobacterales* cases. Carbapenems nonsusceptibility varied among bacterial species.

**Conclusions:**

This study highlights the increasing CR burden of *Acinetobacter*, *P. aeruginosa*, and *K. pneumoniae* in the UAE. Regional variations in resistance, particularly the MDR-CR *Acinetobacter* surge in the NE during the COVID-19 pandemic, stress the need for tailored infection control measures and antimicrobial stewardship.

**Supplementary Information:**

The online version contains supplementary material available at 10.1186/s12879-026-13007-0.

## Background

Carbapenems, critical members of the ß-lactam family, are broad-spectrum antibiotics widely utilized globally. They exert bactericidal effects by inhibiting cell wall biosynthesis and promoting autolysis in Gram-positive, Gram-negative, and anaerobic bacteria, including *Pseudomonas aeruginosa*. Their concentration-independent action, minimal allergic cross-reactions (< 1%), and limited adverse effects- primarily nausea and vomiting (1–20%) and, less frequently, seizures at high doses (1.5%)- render them preferred agents for empirical therapy in severe healthcare-associated infections [1, 2]. Owing to their remarkable stability against most ß-lactamases, carbapenems constitute the first-line treatment for infections caused by extended-spectrum ß-lactamase (ESBL)-producing organisms, thereby contributing to reduced mortality rates [3].

However, the irrational use of carbapenems has led to increasing resistance, especially among Gram-negative bacteria (GNB). Carbapenem-resistant *Enterobacterales* (CRE) (including *Escherichia coli*, *Klebsiella* spp., and *Enterobacter* spp.), carbapenem-resistant *P. aeruginosa* (CRPA), and carbapenem-resistant *Acinetobacter* (CRA) pose significant threats in healthcare settings. These pathogens are implicated in various healthcare-associated infections, including urinary tract infections, intra-abdominal infections, lower respiratory infections, and bloodstream infections. Resistance to carbapenems involves three primary mechanisms: (1) reduced porin permeability, which limits carbapenem influx; (2) efflux pump overexpression, which facilitates carbapenem extrusion; and (3) the production of carbapenemase, an enzyme that hydrolyses and inactivates carbapenems [1, 2]. Carbapenemase-mediated resistance is particularly concerning because of its ability to hydrolyse multiple ß-lactams and often coencodes resistance to other antibiotic classes, such as fluoroquinolones and aminoglycosides. Furthermore, efflux pump overexpression can also lead to multidrug resistance (MDR), as these pumps can expel diverse antibiotics from bacterial cells [1, 2].

In recent decades, carbapenem resistance (CR) has emerged as a significant global clinical and public health concern, with carbapenemase-producing organisms (CPOs) posing considerable threats due to their transmissibility and limited treatment options [4, 5]. Consequently, the World Health Organization (WHO) has ranked these pathogens as “priority 1: critical” [6], whereas the United States Centers for Disease Control and Prevention (CDC) has classified them as “urgent and serious threats” [7], underscoring their paramount public health risk. On a regional level, systematic reviews conducted by Sleiman et al. and Nasser et al. have comprehensively elucidated the increased prevalence of CR in the Arabian Region, synthesizing two decades of research [8, 9]. The United Arab Emirates (UAE) healthcare system faces an escalating burden from antimicrobial resistance (AMR), which prompted the implementation of a National Antimicrobial Resistance Surveillance System in 2015 to monitor antibiotic resistance trends and guide policy decisions [10]. Active surveillance combined with updated treatment guidelines is essential for the early detection and prevention of CR.

Despite ongoing surveillance efforts, comprehensive large-scale studies examining CR across multiple healthcare settings in the UAE remain scarce, with most research limited to single institutions or small cohorts that may not capture the full extent of resistance patterns [11]. Furthermore, little is known about how the COVID-19 pandemic may have influenced resistance trends, despite reports of significant CR outbreaks in other countries during this period [12–14]. This gap underscores the need for extensive studies to assess the current resistance landscape effectively.

This study aims to address these knowledge gaps by determining the prevalence of MDR-CR among GNB isolated from various clinical specimens across different healthcare sectors in Dubai and the Northern Emirates (Sharjah, Ajman, Fujairah, Ras Al Khaimah, and Umm Al Quwain). Additionally, it seeks to phenotypically characterize CR patterns among key pathogens. These findings will inform future antimicrobial stewardship and infection control policies in the UAE, contributing to the global effort to combat AMR.

## Methods

### Study design and setting

This retrospective multicenter study was conducted across 65 healthcare facilities operated by five large healthcare groups (one public and four private) in Dubai and the Northern Emirates (Sharjah, Ajman, Fujairah, Ras Al Khaimah, and Umm Al Quwain) of the UAE between January 2018 and June 2021. The facilities encompassed various levels of patient care, including 11 hospitals (7 in Dubai, 1 in Sharjah, 2 in Ajman, 1 in Fujairah), 49 ambulatory care centers (33 in Dubai, 10 in Sharjah, 3 in Ajman, 1 each in Fujairah, Ras Al Khaimah, and Umm Al Quwain), 3 rehabilitation centers (2 in Dubai and 1 in Ajman), and 2 homecare services (in Dubai) (See Supplementary Table 1).

### Data collection and inclusion/exclusion criteria

**Fig. 1:**
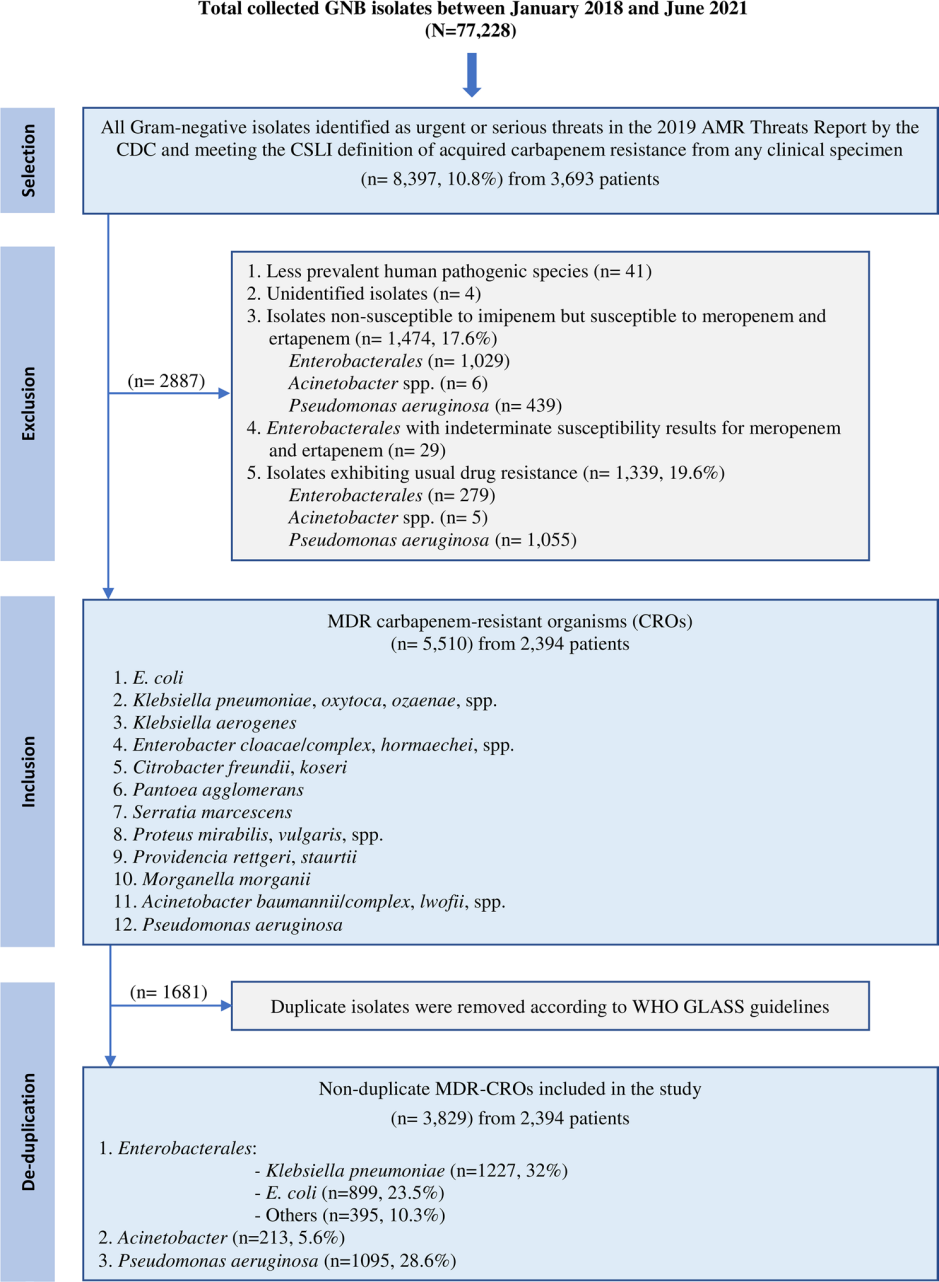
Flowchart illustrating the selection process for the study isolates. Other *Enterobacterales* include *Klebsiella oxytoca* (21)/*ozaenae* (11)/spp. (3), *Klebsiella aerogenes* (58), *Enterobacter cloacae* (93)/*hormaechei* (2)/spp. (12), *Citrobacter freundii* (9)/ *koseri* (45), *Pantoea agglomerans* (11), *Serratia marcescens* (50), *Proteus mirabilis* (53)/ *vulgaris* (3)/spp. (4), *Providencia rettgeri* (4)/*staurtii* (3), and *Morganella morganii* (13). *Acinetobacter* include *Acinetobacter baumannii* (156)/*lwofii* (3)/spp. (54). AMR – antimicrobial resistance, CDC - Centers for Disease Control and Prevention, CROs – carbapenem-resistant organisms, CSLI - The Clinical and Laboratory Standards Institute, GNB – Gram-negative bacteria, MDR - multidrug resistance, WHO GLASS - World Health Organization Global Antimicrobial Resistance and Use Surveillance System

As this was a retrospective study, microbiological testing was performed locally at the participating hospital laboratories in accordance with their routine diagnostic protocols and quality standards, and no additional testing was conducted specifically for this study. Data were extracted electronically from the laboratory information system (LIS), including specimen type, collection date, patient care setting, patient demographics (age, gender, and nationality), identified organism, antibiotic susceptibility testing results (AST)). Identification and antimicrobial susceptibility results were collected for all GNB isolates (*N* = 77,228) obtained from diverse clinical samples (blood, urine, respiratory, wound/pus, biopsy, fluid, catheter tips, and swabs) across all patient settings and age groups throughout the study period (See Supplementary Table 2), which were then analysed to identify CR strains (*n* = 8,397; 10.8%) meeting the Clinical and Laboratory Standards Institute (CLSI) definition of acquired CR (CLSIM100, ED30) [15] and classified as urgent/serious threats in the CDC AMR Threats Report (2019) [7] (Fig. 1). Additionally, MDR strains of clinically significant CRE, CRA, and CRPA with known AST results were segregated on the basis of criteria established by Magiorakos et al. (*n* = 5,510) [16]. Isolates exhibiting usual drug resistance (UDR) [17] and those showing nonsusceptibility to imipenem only [18] were excluded. Data were deduplicated following the World Health Organization Global Antimicrobial Resistance and Use Surveillance System (WHO GLASS; 2021) guidelines, considering only the first isolate per patient, per pathogen, per specimen, per year, per infection origin (*n* = 3,829, 5%) [19]. In instances where multiple isolates of the same bacterial strain from a single patient exhibit varying levels of resistance, the isolate with the highest resistance was selected. Additional LIS variables, including specimen type, collection date, patient setting, and basic demographic information (age, gender, nationality) were extracted for descriptive purposes only.

### Definitions of resistance categories

CR was defined as nonsusceptibility to at least one carbapenem (ertapenem, meropenem, imipenem). For *Proteus* spp., *Providencia* spp., and *Morganella morganii*, which naturally exhibit relatively high minimum inhibitory concentrations (MICs) to imipenem, classification was based on resistance to meropenem and ertapenem [15]. MDR was defined as nonsusceptibility to at least one agent in three or more antibiotic classes [16]. A UDR was defined as nonsusceptibility to carbapenems alone or to only one additional antibiotic class [17].

### Bacterial identification and antibiotic susceptibility testing

Automated microbiological systems (Vitek2 compact, MicroScan, and BD Phoenix-M50) with GNB identification cards were employed for isolate identification and AST across different healthcare settings. For selected isolates, AST confirmation was performed via the disk diffusion and E-test methods. AST results were interpreted using CLSI breakpoints at all the study sites. The variability among systems is acknowledged as a study limitation.

### Statistical analysis

As this was an isolate-level study, prevalence was defined as the proportion of MDR-CR isolates among the total isolates of each bacterial group at a given time point. Prevalence was calculated separately for each bacterial group to identify trends over time (See Supplementary Table 3). The calculations were performed using the following equations:

$$\begin{aligned}&\text{Year-specific prevalence of }\cr & \quad\mathrm{(}\text{type X}\mathrm{)}\text{ MDR-CROs (\%)}\cr & =\left(\frac{\begin{array}{c}\text{}\\\text{}\mathrm{N}\mathrm{u}\mathrm{m}\mathrm{b}\mathrm{e}\mathrm{r}\:\text{}\mathrm{o}\mathrm{f}\:\text{}\mathrm{(}\mathrm{t}\mathrm{y}\mathrm{p}\mathrm{e}\:\text{}\mathrm{X}\mathrm{)}\:\text{}\mathrm{M}\mathrm{D}\mathrm{R}\mathrm{-}\mathrm{C}\mathrm{R}\mathrm{O}\mathrm{s}\\\mathrm{i}\mathrm{n}\:\text{}\mathrm{a}\:\text{}\mathrm{g}\mathrm{i}\mathrm{v}\mathrm{e}\mathrm{n}\text{}\:\mathrm{y}\mathrm{e}\mathrm{a}\mathrm{r}\end{array}}{\begin{array}{c}\mathrm{T}\mathrm{o}\mathrm{t}\mathrm{a}\mathrm{l}\:\text{}\mathrm{n}\mathrm{u}\mathrm{m}\mathrm{b}\mathrm{e}\mathrm{r}\:\text{}\mathrm{o}\mathrm{f}\:\text{}\mathrm{(}\mathrm{t}\mathrm{y}\mathrm{p}\mathrm{e}\:\text{}\mathrm{X}\mathrm{)}\:\text{}\mathrm{i}\mathrm{s}\mathrm{o}\mathrm{l}\mathrm{a}\mathrm{t}\mathrm{e}\mathrm{s}\:\text{}\\\mathrm{i}\mathrm{n}\:\text{}\mathrm{t}\mathrm{h}\mathrm{e}\:\text{}\mathrm{s}\mathrm{a}\mathrm{m}\mathrm{e}\:\text{}\mathrm{y}\mathrm{e}\mathrm{a}\mathrm{r}\end{array}}\right)\cr & \quad\times100\end{aligned}$$

$$\begin{aligned}&\text{Period prevalence of }\cr & \quad\mathrm{(}\text{type X}\mathrm{)}\text{ MDR-CROs (\%)}\cr &=\left(\frac{\begin{array}{c}\mathrm{T}\mathrm{o}\mathrm{t}\mathrm{a}\mathrm{l}\text{}\:\mathrm{n}\mathrm{u}\mathrm{m}\mathrm{b}\mathrm{e}\mathrm{r}\:\text{}\mathrm{o}\mathrm{f}\:\text{}\mathrm{(}\mathrm{t}\mathrm{y}\mathrm{p}\mathrm{e}\:\text{}\mathrm{X}\mathrm{)}\:\text{}\mathrm{M}\mathrm{D}\mathrm{R}\mathrm{-}\mathrm{C}\mathrm{R}\mathrm{O}\\\mathrm{o}\mathrm{v}\mathrm{e}\mathrm{r}\:\text{}\mathrm{t}\mathrm{h}\mathrm{e}\:\text{}\mathrm{s}\mathrm{t}\mathrm{u}\mathrm{d}\mathrm{y}\:\text{}\mathrm{p}\mathrm{e}\mathrm{r}\mathrm{i}\mathrm{o}\mathrm{d}\end{array}}{\begin{array}{c}\mathrm{T}\mathrm{o}\mathrm{t}\mathrm{a}\mathrm{l}\:\text{}\mathrm{n}\mathrm{u}\mathrm{m}\mathrm{b}\mathrm{e}\mathrm{r}\:\text{}\mathrm{o}\mathrm{f}\:\text{}\mathrm{(}\mathrm{t}\mathrm{y}\mathrm{p}\mathrm{e}\:\text{}\mathrm{X}\mathrm{)}\:\text{}\mathrm{i}\mathrm{s}\mathrm{o}\mathrm{l}\mathrm{a}\mathrm{t}\mathrm{e}\mathrm{s}\\\text{}\mathrm{o}\mathrm{v}\mathrm{e}\mathrm{r}\:\text{}\mathrm{t}\mathrm{h}\mathrm{e}\:\text{}\mathrm{s}\mathrm{t}\mathrm{u}\mathrm{d}\mathrm{y}\:\text{}\mathrm{p}\mathrm{e}\mathrm{r}\mathrm{i}\mathrm{o}\mathrm{d}\end{array}}\right)\cr & \quad\times100\end{aligned}$$

Descriptive analyses, including frequency distribution and cross-tabulation, were performed to summarize the resistance characteristics of the isolates. Categorical variables are presented as frequencies and percentages. Data analysis was conducted using IBM SPSS for Windows (v29).

Because the study aimed to evaluate isolate-level burden over time, different counting approaches were applied according to the purpose of the analysis. For prevalence calculations, isolate counts including duplicates were used, with consistency maintained between numerator and denominator. This approach aligns with prior studies demonstrating that the inclusion or exclusion of duplicates does not significantly impact overall prevalence estimates [20]. For descriptive and surveillance-oriented analyses, non-duplicated isolate counts were used to avoid overrepresentation of repeated isolates from the same patient, in line with standard AMR surveillance practices.

## Results

### Proportions of the study collection

A total of 3,829 MDR-CROs were identified across all the study sites. *Enterobacterales* comprised the majority of the isolates (2,521; 65.8%), with *K. pneumoniae* representing 48.7% of this group. *P. aeruginosa* accounted for 1,095 isolates (28.6%) and *Acinetobacter* accounted for 213 isolates (5.6%) (See Supplementary Table 3).

Tertiary care hospitals, particularly in Dubai, bore the highest CR burden, with the two largest tertiary public hospitals collectively accounting for over 56% of all MDR-CROs (See Supplementary Table 1). In contrast, hospitals in the Northern Emirates had a significantly lower share, each contributing less than 3%. Among ambulatory care centers, private facilities in Dubai accounted for 14.9% of MDR-CROs.

### Prevalence of MDR-carbapenem-resistant isolates

**Fig. 2:**
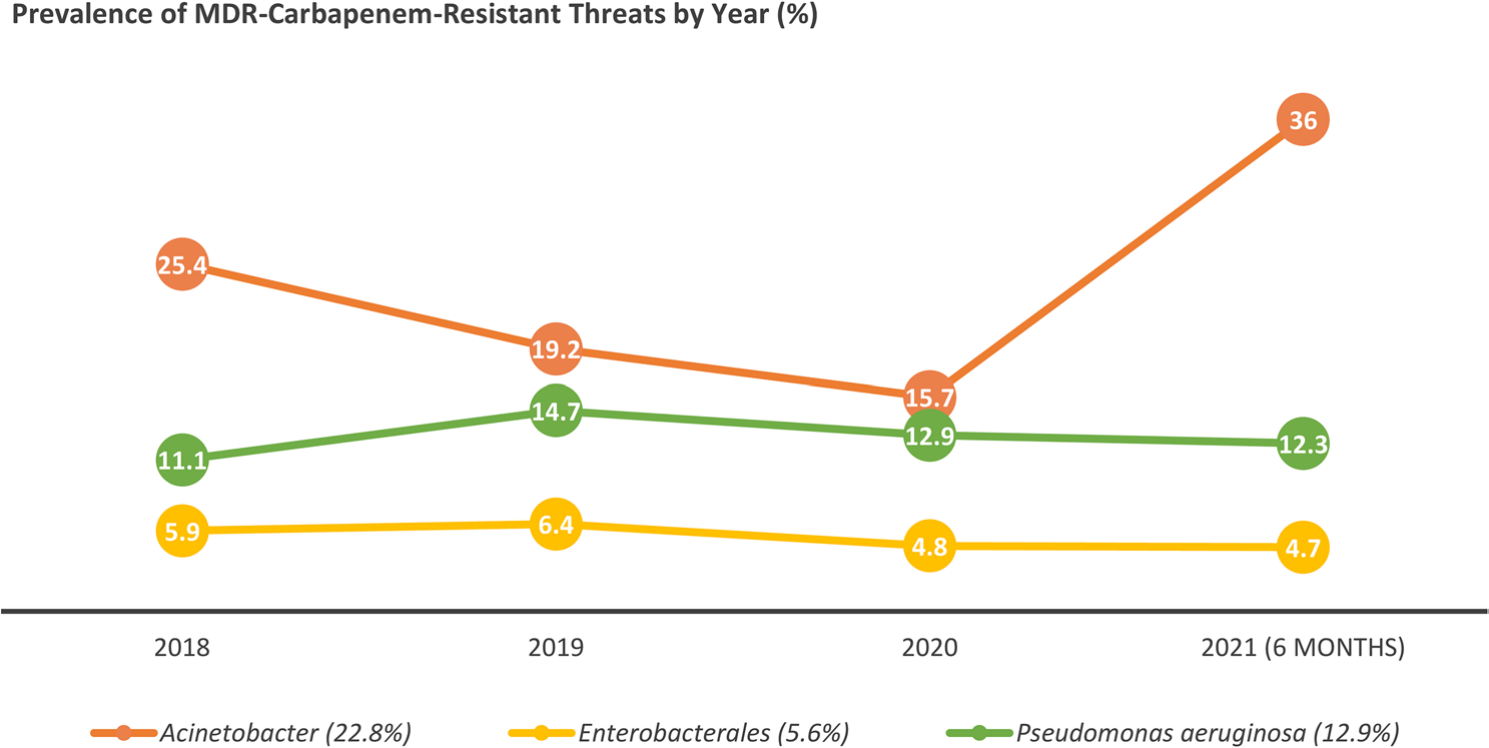
Year-wise trend analysis of carbapenem-resistant threats.The percentages in the plot represent the year-specific prevalence of carbapenem resistance, while those in the legend indicate the period prevalence throughout the study period. Duplicated isolate counts were used for the analysis. *Acinetobacter* (*n* = 315) include *Acinetobacter baumannii*/*lwofii*/spp. *Enterobacterales* (*n* = 3,435) include *E. coli*, *Klebsiella pneumoniae*/*oxytoca*/*ozaenae*/spp., *Klebsiella aerogenes*, *Enterobacter cloacae*/ *hormaechei*/spp., *Citrobacter freundii*/ *koseri*, *Pantoea agglomerans*, *Serratia marcescens*, *Proteus mirabilis*/*vulgaris*/spp., *Providencia rettgeri*/*staurtii*, and *Morganella morganii*. *Pseudomonas aeruginosa* (*n* = 1,760)

Prevalence of MDR-CR among nonfermenting pathogens (*Acinetobacter* and *P. aeruginosa*) consistently exceeded those of fermenting pathogens (*Enterobacterales*) throughout the study period (Fig. 2). The period prevalence was highest for *Acinetobacter* (22.8%; 315/1,380), followed by *P. aeruginosa* (12.9%; 1,760/13,674), and *Enterobacterales* (5.6%; 3,435/61,278).

As illustrated in Fig. 2, MDR-CRA exhibited the highest prevalence, showing a declining trend from 25.4% in 2018 to 15.7% in 2020, followed by a sharp increase to 36% in the first half of 2021, the highest rate observed during the study. MDR-CRPA ranked second, with approximately double the prevalence of MDR-CRE. MDR-CRPA showed a relatively stable trend with minor fluctuations, peaking at 14.7% in 2019 before decreasing slightly to 12.9% in 2020 and remaining almost steady by mid-2021. In contrast, MDR-CRE maintained the lowest prevalence, peaking at 6.4% in 2019 and gradually decreasing to 4.7% by early 2021. Despite these fluctuations, the overall prevalence rates of MDR-CRPA and MDR-CRE remained relatively stable throughout the study, contrasting sharply with the dramatic rise observed in MDR-CRA prevalence during 2021.

### Distribution of MDR-carbapenem-resistant isolates

**Fig. 3:**
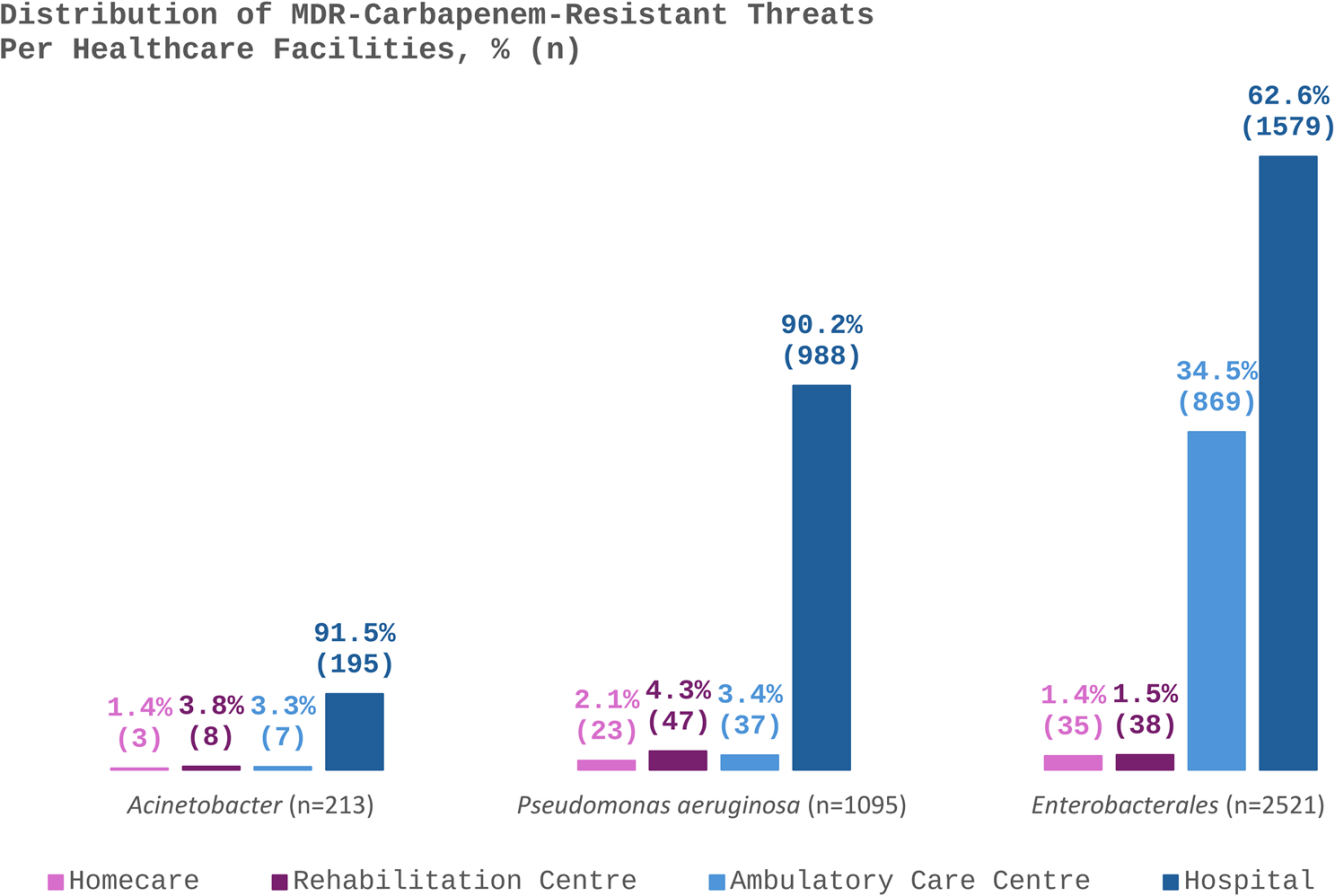
Distribution of MDR-carbapenem-resistant threats according to healthcare facility. Non-duplicated isolate counts were used for the analysis. *Acinetobacter* include *Acinetobacter baumannii* (156)/*lwofii* (3)/spp. (54). *Enterobacterales* include *E. coli* (899), *Klebsiella pneumoniae* (1227)/*oxytoca* (21)/*ozaenae* (11)/spp. (3), *Klebsiella aerogenes* (58), *Enterobacter cloacae* (93)/*hormaechei* (2)/spp. (12), *Citrobacter freundii* (9)/ *koseri* (45), *Pantoea agglomerans* (11), *Serratia marcescens* (50), *Proteus mirabilis* (53)/*vulgaris* (3)/spp. (4), *Providencia rettgeri* (4)/*staurtii* (3), and *Morganella morganii* (13)

The distribution of MDR-CROs across healthcare facilities is shown in Fig. 3. Hospitals were the primary site for both MDR-CRA and MDR-CRPA (> 90%), while MDR-CRE exhibited a broader distribution between hospitals (62.6%) and ambulatory care centers (34.5%). Rehabilitation and homecare centers reported minimal MDR-CROs presence, with rates ranging from 1.4% to 4.3%.

**Fig. 4:**
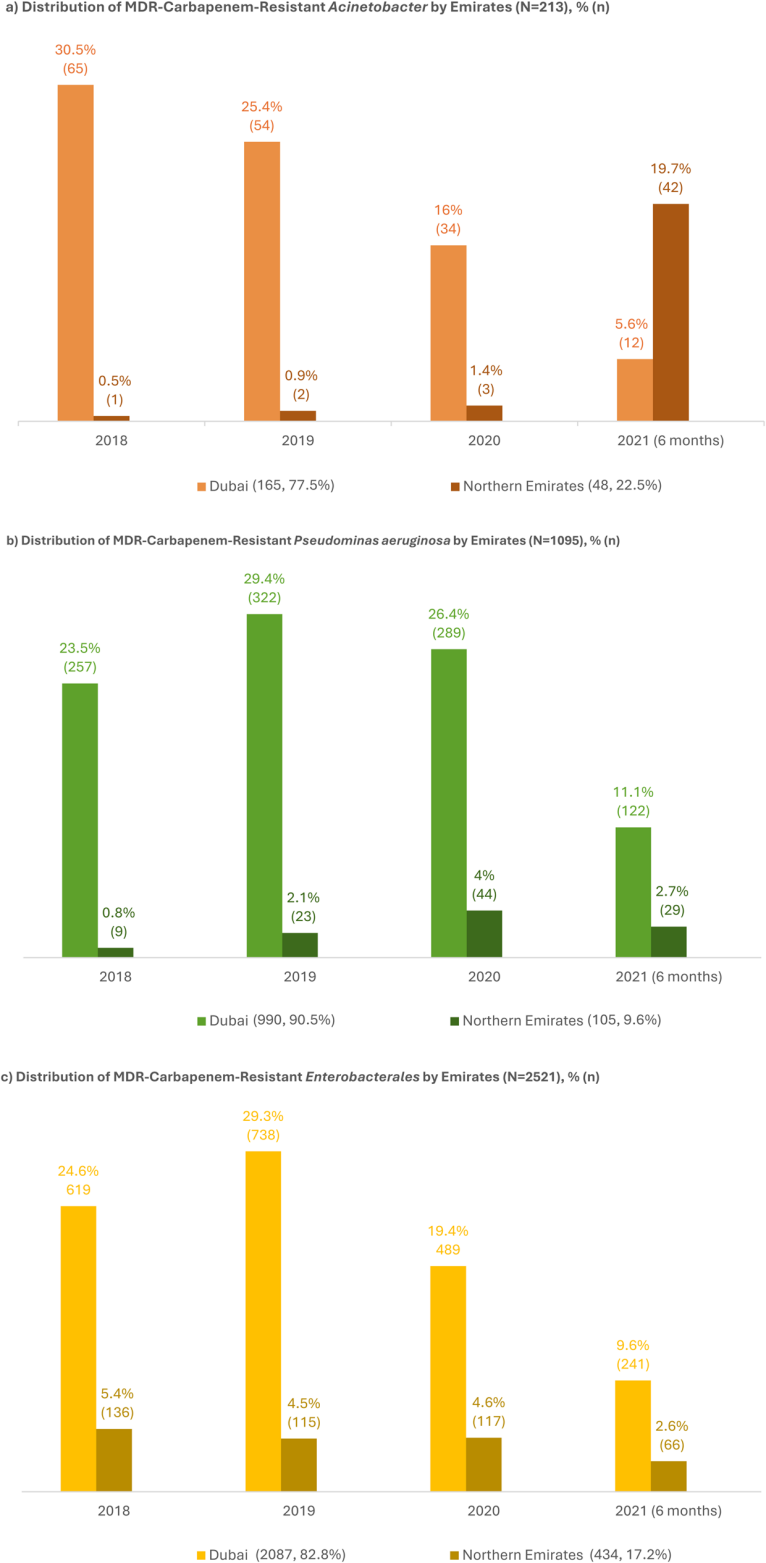
Distribution of MDR-carbapenem-resistant threats according to Emirate. Northern Emirates include Sharjah, Ajman, Fujairah, and Umm Al Quwain. Non-duplicated isolate counts were used for the analysis. *Acinetobacter* include *Acinetobacter baumannii* (156)/ *lwofii* (3)/spp. (54). *Enterobacterales* include *E. coli* (899), *Klebsiella pneumoniae* (1227)/*oxytoca* (21)/ *ozaenae* (11)/spp. (3), *Klebsiella aerogenes* (58), *Enterobacter cloacae* (93)/*hormaechei* (2)/ spp. (12), *Citrobacter freundii* (9)/*koseri* (45), *Pantoea agglomerans* (11), *Serratia marcescens* (50), *Proteus mirabilis* (53)/*vulgaris* (3)/spp. (4), *Providencia rettgeri* (4)/*staurtii* (3), and *Morganella morganii* (13)

Geographical distribution (Fig. 4, a and b, and 4c) revealed that the majority of MDR-CROs were isolated in Dubai (77.5% of MDR-CRA, 82.8% of MDR-CRE, and 90.5% of MDR-CRPA), with underrepresentation from the Northern Emirates (NE) (See Supplementary Table 1). Notably, the MDR-CRA prevalence in NE increased significantly during the first half of 2021- up to 40 fold- from only a few cases in previous years (1–3 cases from 2018 to 2020), whereas a clear downwards trend was observed in Dubai for the same period (Fig. 4a). In contrast, MDR-CRPA and MDR-CRE distributions in NE remained relatively low throughout the study period.

### Phenotypic characteristics of carbapenem resistance

The carbapenem susceptibility testing results for the collected MDR-CROs are summarized in Table 1. *Acinetobacter* and *P. aeruginosa* consistently exhibited high levels of carbapenem resistance, with meropenem resistance exceeding 93% and reaching 98% in *Acinetobacter*, and imipenem resistance surpassing 95% in both organisms.

In contrast, *Enterobacterales* demonstrated a broader range of carbapenem resistance, varying from 57.1% to 100% for ertapenem, 27.3% to 85.7% for meropenem, and 0% to 68.1% for imipenem. Among *Enterobacterales*, *K. pneumoniae* presented the highest resistance to all carbapenems: 95.1% to ertapenem, 82.5% to meropenem, and 68.1% to imipenem. Notably, *Pantoea agglomerans* was completely resistant to ertapenem (100%) but presented the lowest resistance to meropenem (27.3%), with no resistance to imipenem. Conversely, *Providencia* spp. exhibited greater resistance to meropenem (85.7%) than to ertapenem (57.1%), with no isolate susceptible to meropenem.

**Table 1:** Carbapenems susceptibility testing profile of the study isolates (cumulative data, January 2018- June 2021)

Carbapenem-resistant isolates (*N* = 3829)^#^	Ertapenem (*n* = 3792)^*^			Meropenem (*n* = 3824)^*^			Imipenem (*n* = 3710)^*^		
	*R*%	I%	S%	*R*%	I%	S%	*R*%	I%	S%
*E. coli* (*n* = 899)	86	6	8	61.7	14.8	23.5	27.5	26.2	46.3
*Klebsiella pneumoniae* (*n* = 1227)	95.1	3.5	1.3	82.5	5.1	12.5	68.1	19.6	12.3
*Klebsiella species* (*n* = 35)	85.3	11.8	2.9	51.4	5.7	42.9	30.3	24.2	45.5
*Enterobacter species* (*n* = 107)	84.1	13.1	2.8	46.7	3.7	49.5	40	26.7	33.3
*Klebsiella aerogenes* (*n* = 58)	80.4	12.5	7.1	51.7	13.8	34.5	33.3	31.6	35.1
*Citrobacter species* (*n* = 54)	87.8	10.2	2	44.4	13	42.6	17	39.6	43.4
*Serratia marcescens* (*n* = 50)	92	2	6	46.8	2.1	51.1	37.5	31.3	31.3
*Pantoea agglomerans* (*n* = 11)	100	-	-	27.3	27.3	45.5	-	33.3	66.6
*Proteus species* (*n* = 60)	83.9	5.4	10.7	52.5	6.8	40.7	IR	IR	IR
*Morganella morganii* (*n* = 13)	92.3	-	7.7	76.9	-	23.1	IR	IR	IR
*Providencia species* (*n* = 7)	57.1	14.3	28.6	85.7	14.3	-	IR	IR	IR
*Acinetobacter* (*n* = 213)	IR	IR	IR	98.1	1.9	-	95.2	2.9	1.9
*Pseudomonas aeruginosa* (*n* = 1095)	IR	IR	IR	93.2	6.8	-	95.4	1	3.5

^#^Bacterial identification and susceptibility testing were carried out using three automated systems: VITEK 2 Compact (*n* = 2516), BD Phoenix (*n* = 1105), and MicroScan (*n* = 208). For less prevalent bacterial species, susceptibility testing results are reported at the group level, as follows: *Acinetobacter* (including *A. baumannii*, *A. lwofii*, and *A. spp.*), *Citrobacter* species (including *C. koseri* and *C. freundii*), *Enterobacter* species (including *E. cloacae*, *E. hormaechei*, and *E. spp.*), *Klebsiella* species (including *K. oxytoca*, *K. ozaenae*, and *K. spp.*), *Proteus* species (including *P. mirabilis*, *P. vulgaris*, and *P. spp.*), and *Providencia* species (including *P. rettgeri* and *P. staurtii*). Non-duplicated isolate counts were used for the analysis

^*^Susceptibility testing for certain carbapenems were either not performed or not reported for all study isolates; the numbers presented for each carbapenem correspond exclusively to isolates with reported results

I – intermediate, IR - intrinsic resistance, R - resistant, S - susceptible

## Discussion

This retrospective study elucidated the prevalence and carbapenem resistance profile of MDR-CROs across multiple healthcare facilities in Dubai and the Northern Emirates between January 2018 and June 2021. A total of 3,829 clinically significant MDR-CROs were identified, comprising 2,521 MDR-CRE, 213 MDR-CRA, and 1,095 MDR-CRPA. Our findings provide valuable regional insights into the growing public health threat posed by CR-GNB in the UAE, particularly concerning the rise in MDR-CRA during the COVID-19 pandemic surge in the NE.

These results largely corroborate the annual UAE National Surveillance AMR reports, which reported similar trends with minor variations likely attributed to differences in sample size [10, 21–23]. Understanding these local resistance patterns is crucial for developing targeted interventions to mitigate the spread of these pathogens, underscoring the pressing need for tailored infection control protocols and optimized treatment strategies.

### Prevalence of MDR-carbapenem-resistant isolates

Our findings indicate higher prevalence among nonfermenting MDR-CROs, such as *Acinetobacter* and *P. aeruginosa*. This observation aligns with previous studies indicating that these pathogens’ intrinsic resistance to ertapenem, coupled with their capacity to acquire additional resistance mechanisms, underpins their high prevalence [4]. This is broadly supported by systematic reviews by Moghnieh et al. and Sleiman et al., who reported similar trends across Arab countries, mirroring the resistance patterns observed in our study [8, 24]. The high prevalence of MDR-CRA is particularly concerning in critical care settings and among immunocompromised patients, where these organisms cause severe healthcare-associated infections [25].

Interestingly, the relatively stable prevalence of MDR-CRE and MDR-CRPA throughout the study period may imply either controlled outbreaks or persistent levels of resistance. The absence of a significant upwards trend does not diminish their public health importance; hence, continued vigilance through infection control measures and antimicrobial stewardship programs remains essential to manage these entrenched pathogens.

While the overall decline in MDR-CRA prevalence is encouraging, it is important to note the observation of a nosocomial outbreak during the first half of 2021, which temporally coincided with the peak of the COVID-19 pandemic. This overlap is reported as an exploratory observation, without implying causality, and warrants careful consideration given its potential epidemiological relevance. This spike, may be attributed to several factors, including disruptions to standard infection control practices and increased use of invasive devices such as ventilators as well as broad-spectrum antibiotics to manage secondary bacterial infections in COVID-19 patients [26]. Similar MDR-CRA outbreaks have been reported globally during the pandemic, particularly among critically ill patients [27], which underscores the critical importance of robust surveillance and infection control strategies during periods of healthcare system strain.

Unfortunately, molecular characterization of the strains involved in this outbreak was not feasible within this study, because of the lack of routine genomic sequencing at participating healthcare facilities. This limitation precludes our ability to determine whether this surge was due to the emergence of new strains or the spread of existing strains. Additionally, we were unable to follow-up on developments as this occurred at the end of our study period, highlighting the need for continuous monitoring of AMR patterns beyond this study timeframe.

### Distribution of MDR-carbapenem-resistant isolates by healthcare setting

The overwhelming majority (> 90%) of MDR-CROs were isolated from hospital settings, highlighting the persistent challenge posed by these resistant pathogens in healthcare environments. Hospitals provide an ideal environment for the development and dissemination of MDR-CROs because of frequent antibiotic use and the presence of immunocompromised patients. Additional potential risk factors were systematically reviewed by Palacios-Baena et al. (2021) [28].

Our study also revealed a notable prevalence of MDR-CRE in the community setting (34.5%), reflecting concerns about community-associated CRE. This finding is consistent with Kelly et al.’s scoping review, which aimed to elucidate the landscape of community-associated CRE, emphasizing the critical need for targeted control strategies to curb the spread of MDR-CRE [29]. This underscores the urgency of investigating risk factors such as recent hospitalization or travel history, which may drive dissemination beyond healthcare settings. Furthermore, environmental sources may function as reservoirs for MDR-CRO dissemination, necessitating stricter environmental control measures [29, 30].

Although MDR-CROs were less frequently detected in homecare and rehabilitation centers, likely due to reduced exposure to critically ill and high-risk patients, the limited sample size from these settings constrains the generalizability of this observation. Further research utilizing larger databases is warranted to draw more robust conclusions.

### Distribution of MDR-carbapenem-resistant isolates by emirate

From a geographical perspective, our data revealed substantial regional variations, with Dubai accounting for the majority of MDR-CRO isolates. This is likely attributable to the greater number of participating healthcare facilities in Dubai, including major public hospitals that serve larger and more diverse patient populations (Supplementary Table 1). These findings may also reflect differences in patient volume, referral patterns, and infection control practices across regions. Differences in nationality composition, international mobility, and population heterogeneity may also contribute to regional variability in resistance patterns and warrant further investigation (Supplementary Table 2).

A concerning trend was the outbreak of MDR-CRA in facilities within the NE during the COVID-19 surge in mid-2021, in contrast to the steady decline in MDR-CRA cases observed in Dubai during the same period. This increase was primarily noted in hospitals located in Sharjah and Ajman, which may reflect facility-specific challenges, local environmental conditions, or preexisting epidemiological factors that predispose these facilities to MDR-CRA outbreaks (as suggested by professionals from the participating facilities). Potential contributing factors may include the presence of local bacterial strains with relatively high resistance profiles or historical patterns of antimicrobial use. Additionally, frequent interhospital patient transfers necessitated by bed shortages during the COVID-19 pandemic, particularly from high-burden areas or facilities with limited ICU capacity, could have facilitated the regional spread of resistant strains [31]. Although preliminary reports suggest a decline in MDR-CRA cases in NE facilities following the study period, continued surveillance is necessary to validate whether this observed decline represents a sustained reduction in MDR-CRA prevalence.

In contrast, the prevalence rates of MDR-CRPA and MDR-CRE in the NE region during mid-2021 remained relatively low, at 2.7% and 2.6%, respectively. This suggests that while MDR-CRA pose a significant challenge in the region, MDR-CRPA and MDR-CRE are either better controlled or inherently less prevalent. Unfortunately, a direct comparison with national AMR data, which encompass a larger and more comprehensive dataset, was not feasible because of the absence of the 2021 national report. The reasons behind this regional disparity, particularly regarding differences in healthcare practices, infection control measures, and the regional impact of COVID-19, remain hypothesis-generating and warrant further investigation in purpose-designed studies to assess whether any association or causal relationship exists between the pandemic and AMR.

### Phenotypic characteristics of carbapenem resistance

The observed discrepancies in CR profiles among MDR-CROs likely stem from diverse resistance mechanisms, including carbapenemase production and other nonenzymatic pathways [2]. However, these data should be interpreted cautiously because of potential methodological limitations imposed by the use of different AST systems (Vitek2, BD Phoenix, and MicroScan), which may influence the MIC results and compromise comparability. Each system exhibits unique performance characteristics concerning sensitivity and specificity [32]. Comparative studies using standardized AST methods would help mitigate this limitation and enhance data reliability.

Overall, our findings highlight the substantial heterogeneity of CR within *Enterobacterales*, in contrast with the more consistent resistance patterns observed in nonfermenters such as *Acinetobacter* and *P. aeruginosa*. The increased resistance to ertapenem is particularly noteworthy, as it often serves as a sentinel marker for CPOs, suggesting a need for rigorous screening protocols in hospital settings [33]. The alarmingly high resistance rates observed in *K. pneumoniae* across all carbapenems signal an urgent need for alternative therapeutic options. This finding aligns with reports from multiple countries across Europe, South and East Asia, North America, Africa, and Russia, where carbapenemase production is prevalent. These global trends are comprehensively summarized in the review by Ma et al., which highlights recent advances in understanding CRE [34]. This variability in resistance patterns significantly complicates the selection of empiric antibiotic therapies and further challenges clinical management strategies in regions where CR pathogens are endemic.

From a clinical perspective, these findings underscore the importance of implementing tailored infection control measures and highlight the urgency of expanding therapeutic options against MDR-CROs. The limited treatment options available to combat these organisms often force clinicians to rely on more toxic alternatives, such as colistin, which pose significant risks to patients [35].

### Limitations

This study has several limitations that warrant consideration. First, the absence of genomic analysis restricts our ability to identify the precise mechanisms driving CR. Second, the geographical concentration of isolates from Dubai, owing to higher representation, may potentially affect the generalizability of the findings to other emirates. Third, given the isolate-based design of this retrospective study, patient-level demographic factors were not analyzed in depth, which may limit assessment of the potential influence of population heterogeneity on regional resistance patterns. Fourth, the reliance on diverse AST systems without interlaboratory standardization may introduce variability in the reported resistance rates. Fifth, the study lacked data on antibiotic usage and infection-control bundle compliance. Finally, the study’s temporal boundaries constrain our ability to capture poststudy trends, thereby limiting our assessment of long-term resistance patterns following the COVID-19 pandemic. These limitations emphasize the need for ongoing surveillance, expanded multicenter studies, and standardized methodologies to enhance infection control strategies and inform evidence-based antimicrobial stewardship programs at the regional level.

## Conclusions

This study highlights the growing threat posed by MDR-CROs in the UAE, with a particularly troubling rise in MDR-CRA during the COVID-19 pandemic in the Northern Emirates. This raises significant concerns regarding the impact of pandemics on healthcare-associated infections and potential lapses in infection control measures. These findings call for targeted infection prevention and control measures in different regions, coupled with continuous monitoring to curb the spread of these virulent pathogens. Strengthening rational antibiotic use, along with finding new treatment options, is essential to address this growing public health challenge. Without prompt and decisive action, the looming “postantibiotic era”, where common infections may become untreatable, is a real threat. Further molecular and epidemiological research into the mechanisms of resistance acquisition and transmission will be key to shaping future strategies and policies. Moreover, the establishment of well-coordinated, multitiered strategies encompassing local, regional, and national levels is crucial. Enhanced collaboration with global AMR surveillance networks will facilitate data sharing and enable the adaptation of successful interventions from other regions.

## Supplementary Information

Below is the link to the electronic supplementary material.

Supplementary Material 1

Supplementary Material 2

Supplementary Material 3

## Data Availability

The datasets generated and analysed during this study were used under license and therefore not publicly available owing to binding data-sharing agreements with the participating hospitals. However, they may be obtained from the corresponding author upon reasonable request and with permission from the participating hospitals.

## Abbreviations

AMR: Antimicrobial Resistance
AST: Antimicrobial Susceptibility Testing
CDC: Centers for Disease Control and Prevention
CLSI: The Clinical and Laboratory Standard Institute
CPO: Carbapenemase Producing Organism
CR: Carbapenem Resistance
CRA: Carbapenem-Resistant Acinetobacter
CRE: Carbapenem-Resistant Enterobacterales
CRO: Carbapenem-Resistant Organism
CRPA: Carbapenem-Resistant Pseudomonas aeruginosa
ESBL: Extended-Spectrum Beta-Lactamase
GLASS: Global Antimicrobial Resistance and Use Surveillance System
GNB: Gram-Negative Bacteria
MDR: Multidrug Resistance
MIC: Minimum Inhibitory Concentration
NE: Northern Emirates
UAE: United Arab Emirates
UDR: Usual Drug Resistance
WHO: World Health Organization
