# Supplementary material for: Prevalence and phenotypic characterization of carbapenem resistance in multidrug-resistant Gram-negative bacteria across selected healthcare facilities in the United Arab Emirates: a retrospective study
Source: BMC Infect Dis. 2026 Mar 13;26:804. doi: 10.1186/s12879-026-13007-0 (PMC13101191; doi:10.1186/s12879-026-13007-0)
Supplement: Supplementary file 1 — Supplementary Material 1 [file 12879_2026_13007_MOESM1_ESM.docx]

**Supplementary Table 1.** Characteristics of the participating healthcare facilities

| **Facility Type**  **(n)^*^** | **Region** | **Sector** | **Category** | **Level of**  **Care^**^** | **Beds^***^** | **% (n) in**  **MDR-CROs^****^** |
| --- | --- | --- | --- | --- | --- | --- |
| **Hospitals (11)** | | | | | | |
| **grpA-H1** (1) | DXB | Public | General Hospital | Tertiary | >500 | **36.2%** (1388) |
| **grpA-H2** (1) | DXB | Public | General Hospital | Tertiary | >500 | **20.3%** (777) |
| **grpA-H3** (1) | DXB | Public | Maternity & Pediatric Hospital | Tertiary | 300-500 | **1.3%** (49) |
| **grpA-H4** (1) | DXB | Public | General Hospital | Secondary | 50-100 | **1.7%** (66) |
| **grpB-H** (1) | DXB | Private | General Hospital | Secondary | 100 | **6.1%** (234) |
| **grpC-H** (1) | DXB | Private | General Hospital | Tertiary | 50-100 | **0.9%** (34) |
| **grpD-H1** (1) | DXB | Private | General Hospital | Secondary | 100-200 | **0.3%** (10) |
| **grpD-H2** (1) | AJM | Private | General Hospital | Secondary | 200-300 | **1.6%** (60) |
| **grpD-H3** (1) | AJM | Private | University Hospital | Tertiary | 300-500 | **0.9%** (36) |
| **grpD-H4** (1) | FUJ | Private | General Hospital | Secondary | 50-100 | **0.4%** (14) |
| **grpE-H** (1) | SHJ | Private | General Hospital | Tertiary | 100-200 | **2.5%** (94) |
| **Ambulatory Care Centers (49)** | | | | | | |
| **grpA-OP** (14) | DXB | Public | Polyclinics | Primary and Secondary | - | **0.8%** (30) |
| **grpB-OP1** (13) | DXB | Private | General & Polyclinics  Corporate Medical Services |  | - | **14.6%** (559) |
| **grpB-OP2** (4) | SHJ | Private | Polyclinics |  | - | **6.7%** (256) |
| **grpB-OP3** (1) | AJM | Private | Polyclinic |  | - | **1%** (40) |
| **grpC-OP** (3) | DXB | Private | Polyclinics  Day Surgery Center |  | - | **0.2%** (9) |
| **grpD-OP1** (3) | DXB | Private | Polyclinics |  | - | **0.03%** (1) |
| **grpD-OP2** (6) | SHJ | Private | Polyclinics  Daycare Hospitals |  | - | **0.3%** (10) |
| **grpD-OP3** (2) | AJM | Private | Polyclinics |  | - | **0.1%** (3) |
| **grpD-OP4** (1) | FUJ | Private | Polyclinic |  | - | **0** |
| **grpD-OP5** (1) | RAK | Private | Polyclinic |  | - | **0** |
| **grpD-OP6** (1) | UMQ | Private | Polyclinic |  | - | **0.2%** (6) |
| **Rehabilitation Centers (3)** | | | | | | |
| **grpC-Rehab** (2) | DXB | Private | Convalescence Houses | Primary | 22-28 | **0.6%** (24) |
| **grpD-Rehab** (1) | AJM | Private | Rehabilitation Hospital | Primary | 100 | **1.8%** (68) |
| **Homecare Centers (2)** | | | | | | |
| **grpA-HC** (1) | DXB | Public | Home Healthcare Services | Primary | - | **1.2%** (45) |
| **grpB-HC** (1) | DXB | Private | Home Healthcare Services | Primary | - | **0.4%** (16) |

* The study was conducted across 65 healthcare facilities of varying categories, operated by five major healthcare groups; one public (grpA; n=19) and four private (grpB; n=20, grpC; n=6, grpD; n=19, grpE; n=1). These facilities are

distributed throughout Dubai and the Northern Emirates (Sharjah, Ajman, Fujairah, Ras Al Khaimah, and Umm Al Quwain) as presented in the table above.

** This classification was based on self-designation obtained from official hospital websites or direct communication.

*** The number of hospital beds was sourced from the Dubai Annual Health Statistics Book (DHA open data; <https://www.dha.gov.ae/en/open-data> for Dubai facilities and from official hospital websites for facilities in the

Northern Emirates where official centralized statistics were not publicly available.

**** A total of 3829 MDR-CR isolates were collected from all participating healthcare groups, distributed as follows: grpA (2355), grpB (1105), grpC (67), grpD (208), grpE (94), with the majority originating from grpA and grpB.

MDR-CROs – Multidrug resistant carbapenem resistant organisms, DXB - Dubai, SHJ – Sharjah, AJM – Ajman, FUJ – Fujairah, RAK – Ras Al Khaimah, UMQ – Umm Al Quwain.
