# Supplementary material for: Prevalence and phenotypic characterization of carbapenem resistance in multidrug-resistant Gram-negative bacteria across selected healthcare facilities in the United Arab Emirates: a retrospective study
Source: BMC Infect Dis. 2026 Mar 13;26:804. doi: 10.1186/s12879-026-13007-0 (PMC13101191; doi:10.1186/s12879-026-13007-0)
Supplement: Supplementary file 2 — Supplementary Material 2 [file 12879_2026_13007_MOESM2_ESM.docx]

**Supplementary Table 2.** Basic Characteristics of the study isolates

| **Parameters** | **n (%) in Study MDR-CROs (n=3829)** |
| --- | --- |
| **Isolated Pathogens^*^** | |
| *Acinetobacter baumannii* | 82 (2.1%) |
| *Acinetobacter baumannii complex* | 74 (1.9%) |
| *Acinetobacter lwoffii group* | 3 (0.1%) |
| *Acinetobacter* | 54 (1.4%) |
| ***Pseudomonas aeruginosa*** | **1095 (28.6%)** |
| ***Klebsiella pneumoniae*** | **1227 (32%)** |
| *Klebsiella aerogenes* | 58 (1.5%) |
| *Klebsiella oxytoca* | 21 (0.6%) |
| *Klebsiella ozaenae* | 11 (0.3%) |
| *Klebsiella species* | 3 (0.1%) |
| ***E. coli*** | **899 (23.5%)** |
| *Enterobacter cloacae* | 71 (1.8%) |
| *Enterobacter cloacae complex* | 22 (0.6%) |
| *Enterobacter hormaechei* | 2 (0.1%) |
| *Enterobacter* | 12 (0.3%) |
| *Citrobacter freundii* | 9 (0.2%) |
| *Citrobacter koseri* | 45 (1.2%) |
| *Serratia marcescens* | 50 (1.3%) |
| *Morganella morganii* | 13 (0.3%) |
| *Proteus mirabilis* | 53 (1.4%) |
| *Proteus vulgaris* | 3 (0.1%) |
| *Proteus* | 4 (0.1%) |
| *Providencia rettgeri* | 4 (0.1%) |
| *Providencia staurtii* | 3 (0.1%) |
| *Pantoea agglomerans* | 11 (0.3%) |
| **Clinical Specimens** | |
| Blood | 276 (7.2%) |
| **Urine** | **1676 (43.8%)** |
| **Respiratory samples** | **839 (22%)** |
| **Wound/Pus** | **739 (19.3%)** |
| Biopsy (tissue/bone)^**^ | 70 (1.8%) |
| Body Fluid/CSF^**^ | 58 (1.5%) |
| Swabs (mouth/ear/eye/genital)^**^ | 142 (3.7%) |
| Catheter Tips | 29 (0.8%) |
| **Patient Setting** | |
| Emergency | 292 (7.6%) |
| **Outpatient** | **1206 (31.5%)** |
| **Inpatient (Non-Intensive Care Unit)** | **1349 (35.2%)** |

| **Inpatient (Intensive Care Unit)** | **982 (25.6%)** |
| --- | --- |
| **Carbapenemase Production** | |
| **Yes** | **1201 (31.4%)** |
| No | 874 (22.8%) |
| **Not screened** | **1754 (45.8%)** |
| **Patient Demographics***** | |
| **Male** | **2053 (53.6%)** |
| Female | 1776 (46.4%) |
| Emirati | 1102 (28.8%) |
| **Non-Emirati^****^** | **2727 (71.2%)** |
| Neonates (<1 month) | 14 (0.4%) |
| Children (>1 month – 14 years) | 287 (7.5%) |
| **Young-aged Adults (15-44 years)** | **1211 (31.6%)** |
| **Middle-aged Adults (45-64 years)** | **909 (23.7%)** |
| **Old-aged adults (65+ years)** | **1408 (36.8%)** |

^*^ Study isolates were categorized into three primary pathogen groups: *Acinetobacter* (n=213), *Pseudomonas aeruginosa*

(n=1095), and *Enterobacterales* (n=2521).

^**^ Among biopsy and fluid samples, only one isolate was obtained from bone marrow and two from cerebrospinal fluid, while the majority of swabs were collected from ear (n=117).

^***^ Demographic data are reported at the isolate level, reflecting the patients from whom isolates were cultured at the time of specimen collection. Consequently, individual patients may contribute multiple isolates due to recurrent infections, different specimen sources, or repeated sampling over time. Patient level de-duplication was not performed.

^****^ The largest proportion of study samples originated from patients of the Arabian Peninsula (n=1256) and the Indian Subcontinent (n= 1251), followed by the Middle East (n=441), Africa (n=408), South and East Asia (n=321), and other regions (n=152).
