# Supplementary material for: Prevalence and phenotypic characterization of carbapenem resistance in multidrug-resistant Gram-negative bacteria across selected healthcare facilities in the United Arab Emirates: a retrospective study
Source: BMC Infect Dis. 2026 Mar 13;26:804. doi: 10.1186/s12879-026-13007-0 (PMC13101191; doi:10.1186/s12879-026-13007-0)
Supplement: Supplementary file 3 — Supplementary Material 3 [file 12879_2026_13007_MOESM3_ESM.docx]

**Supplementary Table 3.** Comprehensive year-wise distribution of Gram-negative bacterial isolates from participating healthcare facilities (January 2018 - June 2021)

| **Organism** | **Year** | | | | **Sum** |
| --- | --- | --- | --- | --- | --- |
|  | **2018** | **2019** | **2020** | **mid-2021** |  |
| **Total GNB (CROs & CSOs)** | 22395 | 23911 | 20614 | 10308 | 77228 |
| **Total duplicated MDR-CROs** | 1574 | 1889 | 1356 | 691 | **5510** |
| **Total de-duplicated MDR-CROs** | 1087 | 1254 | 976 | 512 | **3829** |
|  | | | | | |
| **Total *Enterobacterales*** | 17873 | 18825 | 16266 | 8314 | 61278 |
| **Total duplicated MDR-CRE** | 1059 | 1198 | 787 | 391 | **3435** |
| **Total de-duplicated MDR-CRE** | 755 | 853 | 606 | 307 | **2521** |
|  | | | | | |
| **Total *Acinetobacter*** | 394 | 401 | 357 | 228 | 1380 |
| **Total duplicated MDR-CRA** | 100 | 77 | 56 | 82 | **315** |
| **Total de-duplicated MDR-CRA** | 66 | 56 | 37 | 54 | **213** |
|  | | | | | |
| **Total *Pseudomonas aeruginosa*** | 3747 | 4170 | 3991 | 1766 | 13674 |
| **Total duplicated MDR-CRPA** | 415 | 614 | 513 | 218 | **1760** |
| **Total de-duplicated MDR-CRPA** | 266 | 345 | 333 | 151 | **1095** |

CRA - carbapenem-resistant *Acinetobacter*, CRE - carbapenem-resistant *Enterobacterales*, CROs – carbapenem- resistant organisms, CRPA - carbapenem-resistant *Pseudomonas aeruginosa*, CSOs - carbapenem-sensitive organisms, GNB - Gram- negative bacteria, MDR – multidrug resistant.
